# Developing a virtual mock casc for trainees

**DOI:** 10.1192/bjo.2021.440

**Published:** 2021-06-18

**Authors:** Laura Somerville, Peter McMurray, Vivian Sing, Stephanie Campbell, Meta McGee

**Affiliations:** 1 South Eastern HSCT, Northern Ireland Medical and Dental Training Agency; 2 Western HSCT, Northern Ireland Medical and Dental Training Agency; 3 Southern HSCT, Northern Ireland Medical and Dental Training Agency; 4 Northern HSCT, Northern Ireland Medical and Dental Training Agency; 5 University of Hull, Northern Ireland Medical and Dental Training Agency

## Abstract

**Aims:**

The restrictions experienced due to the COVID-19 pandemic had impacts on how clinical teaching and assessment is conducted. The Royal College of Psychiatrists decided to run the final part of the membership exam, the Clinical Assessment of Skills and Competencies (CASC) online for the first time in September 2020. We aimed to prepare candidates in the Northern Ireland deanery for this by developing a virtual mock examination using the Zoom platform.

**Method:**

In previous years, higher psychiatry trainees in the Northern Ireland deanery have run successful face to face mock examinations to help pre-membership trainees prepare for the CASC. We adapted some of this material to our virtual examination. 16 stations were run in total, in two circuits of eight. These stations were mapped to the Royal College CASC blueprint. Higher trainees were recruited to act as examiners, with core trainees acting as simulated patients. The mock examination was advertised through the local deanery and all candidates sitting in September availed of the opportunity (a total of 8 trainees).

Zoom was used as the platform due to ease of use, familiarity and breakout room function. Each station formed one breakout room, and a facilitator moved candidates between rooms and provided timing prompts. Instructions were emailed to candidates in advance.

A comfort break was provided between circuits. At the end of the mock examination, everyone was returned to the main room and examiners gave general feedback and tips. Individual feedback was provided by collating mark schemes for each candidate, which included free text feedback, and sending these via email.

**Result:**

Despite the evident challenges involved, the mock CASC ran smoothly. There was one minor delay of approximately 3 minutes due to technical difficulties, which was easily recouped. We obtained qualitative feedback from candidates which was positive, with trainees commenting that they felt “more at ease … less worried” about a digital exam, and that it was “efficient and effective”.

All candidates who sat the mock examination were successful in the face to face CASC sitting which followed in September.

**Conclusion:**

We were able to successfully adapt what was previously an in-person mock CASC exam to the new digital format in a way that reflected how the actual CASC exam will run, and it was considered beneficial preparation by the candidates who sat this mock. This has improved trainee experience at a time when many teaching opportunities have been suspended.

